# Army News

**Published:** 1918-08

**Authors:** 


### ARMY NEWS.

It's a splendid report that Dr. G. B. Foscue, chairman of the auxiliary committee, medical defense for McLennan county, has sent to the state chairman, and the following brief in the report has been called to the attention of Dr. Franklin Martin, chairman of the advisory committee, Council of National Defense, Washington:

There were 152 registered physicians in McLennan county: 30 are now in the active service of the army; 40 are over 55 years of age; 23 are between the ages of 31 and 55 and were put in class 1; 36 are between the ages of 31 and 55 and were put in classes 2 and 3; 8 have passed their examination and are awaiting commissions; 4 have been officially rejected; 2 have been commissioned and are now awaiting orders; 1 was discharged from service on account of disability; 6 women physicians in the county.

Out of a total o fthe eligible physicians of the county, between the ages of 31 and 55, 68 in number, 45 are either in the army or navy, or have volunteered their services. There are only four in the county who are within the draft age. One of these is waiting a commission, and one has entered the United States public health service.

This is the final report of the auxiliary committee, members of which have completed their work.—Waco Times-Herald.

Lieut. William H. Cade of San Antonio, is with the Reserve Expeditionary Forces in France in the Medical Reserve Corps. He completed the course in X-Ray work required by the Government at Cornell University and will follow that line of work while in the service in France.

Dr. Joe E. Dildy, who was recently commissioned first lieutenant in the army medical reserve corps, has been ordered to Fort Sam Houston at San Antonio for assignment to duay.

Dr. J. J. Taubenhous of the A. and M. College has been appointed official representative of the Jewish welfare board of the United States Army and Navy . This board has been approved officially by Secretaries Baker and Daniels. Its activities are within the camps along the same lines as the Y. M. C. A. and other religious organizations.

First Lieutenants Charles M. McMillan and W. D. Hardesty of the medical reserve corps, have reported to the adjutant at Camp McArthur for duty there. These officers have just completed a course in the medical officers' training camp at Fort Oglethrope, Ga.

Dr. W. C. Tennery, of Waxahachie, who has volunteered in the Medical Reserve Corps, has been notified of his appointment as first lieutenant.

Dr. John T. Waston, president of the Dallas County Medical Society, was on June 12 commissioned Captain in the Medical Reserve Corps. He is subject to call.

Dr. Garrett Hogg and Dr. R. W. Wells, both of Edna, have enlisted in the reserve medical corps of the United States army and expect to be called into service in the near future. The former has been county health officer of Jackson County for several years, while the latter has been a member of the local exemption board since it was organized. Both are men with families and are over the draft age.

Dr. Jack Saunders of Fort Worth, who has recently volunteered and joined the Medical corps of the U. S. Army, has received his commission as First Lieutenant, and has been ordered to report for duty in fifteen days.

Dr. Walter Brown of Beaumont, has received a commission as lieutenant in the United States army and has been ordered to Camp Logan, Houston.

Dr. W. C. Duringer, one of the best known young physicians and surgeons in Fort Worth, who volunteered his services to the government several weeks ago, has been commissioned as a captain in the medical reserve corps.

Caldwell County has three of her physicians now in France, and a dozen or more in the various army cantonments. Dr. Lan L. Hewlett, who went with the 36th Division, has sailed and will then be with Dr. Cranz Nicholl of Maxwell and Dr. Fletcher Clark, who went over with the 90th Division. All three are captains.

Dr. A. J. Turner of Beeville has received his commission as a first lieutenant in the officers' medical reserve corps of the national army. Dr. Turner is the sixth Beeville practitioner to receive a commissions in the army.

Capt. P. D. Barnhill of Brenham, recently commissioned in the United States Medical Corps, has been ordered to report at Fort Sam Houston.

Dr. McCrum of Lone Oak, Texas, volunteered his services to the government and the department has wired him his commission. Dr. McCrum has a son, Lieut. Douglas McCrum, who is instructor at Hicks Field, Ft. Worth.

Dr. F. H. Newton of Dallas, who volunteered his services in the Medical Reserve Corps about two months ago, has been called for active duty in the base hospital at Camp Logan, Houston.

First Lieut. Samuel D. Weaver of De Leon, Texas, has arrived safely overseas, probably in Italy, according to messages received by his parents, Dr. and Mrs. T. P. Weaver. In July, 1917, he was elected to Fellowship at Mayo's Rochester, Minn., and served there for some months, then tendered his services to the government. He was commissioned a first lieutenant Jan. 3, 1918, and sent to Fort McPherson, Ga., to train with the Mayo unit. He was transported the early part of this month.

Dr. Carl A. Dreiss of Fort Worth, Texas, has received appointment from Washington as first lieutenant in the Medical Reserve Corps.

A number of the friends of Dr. I. P. Sessions of Rockdale, Texas, tendered him a "fish fry" at Club Lake. The affair was in the nature of a farewell to Dr. Sessions, who left for Fort Sam Houston to report for duty; he having been commissioned as captain in the medical reserve corps. A number of toasts were offered during the progress of the banquet which were feelingly responded to by both Captain Sessions and Mrs. Sessions.

Among the reports of commissions issued in the United States army recently was that of Dr. E. A. Benbow of Luling Springs, Texas, who receives a first lieutenancy in the medical department.

Dr. J. B. Hester of Itasca, Texas, received orders last week to report to New Orleans for examination in the medical branch of the United States navy, in which he volunteered his services some time ago.

Relatives in Duncan, Okla., of Lieut. John A. White, have received word that he is being held a prisoner in a German camp, but the exact location is not known. White's boyhood home was in Clarkesville, Texas, though with his mother and other relatives he removed from there to Philadelhpia, where he graduated from a medical college. He at first enlisted in the British hospital corps, but later was transferred to an American regiment.

Captain Isaac Arnold Withers, M. D., of Fort Worth, having received his orders, will report at Camp Greenleaf, Fort Oglethrope. Captain Withers volunteered for service several weeks ago and received his commission shortly after volunteering.

Lieut. C. B. Gant of Graham, Texas, is now serving in the Medical Corps of the United States army in France. Soon after this country entered the war, Dr. Gant volunteered for service and was accepted. He was called into service last January, served at Camp Travis for a short time and was then sent across.

Five medical officers arrived at the Camp Bowie base hospital recently from California. They were: Capt. Edward H. Jordan, Presidio; Capt. John A. Mapes, Presidio; Capt. S. M. Alter, San Francisco; First Lieut. William S. McCurray, San Francisco; First Lieut. Oliver W. Butler, San Francisco. First Lieut. Holman Bernard has been transferred from the base hospital to camp headquarters for duty.

Major Edgar W. Loomis of the Medical Reserve Corps, and formerly city health officer of Dallas, Texas has landed in France and now is serving with a number of Dallas men who are headed for Berlin. Word has been received that Major Loomis now is close to the firing line.

Major Loomis was head of the city health department for more than two years but resigned when the first call came to arms. He was a captiain in the old Texas National Guard and his training in the guard gave him many advantages. Captain Loomis was promoted to the rank of major. He was stationed at San Antonio with the Third Field Artillery for several months and then sent to Fort Sill, Okla., but left there about a month ago and was sent overseas.

Dr. William Lucy, of Cleburne, Texas, cabled his wife and mother that he had arrived safely overseas. He is in the hospital service with the American expeditionary forces.

Dr. J. L. Borden, of Victoria, Texas, has received a commission of first lieutenant in the United States Medical Corps.

Dr. A. M. McElhannon, of Sherman, Texas, has been commissioned as a captain by the war department.

Dr. O. J. Cook, of Laredo, but for some months a practicing physician at Potrerillos, Chile, was recently commissioned first lieutenant in the Medical Reserve Corps. He is a graduate of Baylor Medical College, Dallas, with the class of 1912, and was with the Pershing expedition in Mexico.

Dr. John Oliver McReynolds, former dean of the Southwestern University Medical School at Dallas, was recently examined and recommended for appointment in the Medical Reserve Corps.

Dr. J. R. Middlebrook, of Alpine, Texas, went to El Paso recently where he placed his application with the War Department for overseas serpice. The doctor had been previously rejected on account of the glass eye but he has appealed his case to Surgeon General Gorgas and believes that he will be accepted.

Dr. C. P. Cook, a well-known Ennis physician, has received a message announcing that he has been accepted for war service in the Medical Reserve Corps with the rank of Captain, and he is now awaiting orders from the Surgeon General.

Dr. Frank Pierce, physician member of the East Dallas Draft Board has been commissioned a Captain in the Medical Reserve Corps, according to notice received from Washington. He will continue with the draft board until called to actual service.

Capt. R. H. Kuhns, medical corps, Carruthers field, has been named post surgeon to succeed Capt. C. E. Campbell, who has been transferred to Mineola, L. I.

Captain Kuhns was one of the first medical officers to come to Ft. Worth when the fields were open. He has seen foreign service. For eight months he was surgeon in an American Red Cross hospital in Russia. When relieved of duty there he returned to America through Germany.

Capt. C. H. Cogswell of Cedar Rapids, Iowa, senior surgeon of Camp Dick, recently received his commission as major in the medical corps. He is quite a distinguished surgeon of national and international repute. During 1915 and 1916 he served as surgeon and ambulance driver in France.

Three Dallas physicians now in France, Drs. C. H. Standifer, W. W. Shortal and R. S. McBride, have been promoted to the rank of Captain, according to a letter just received by Mrs. Standifer from her husband. All are in the medical service.

Overcoats and heavy clothing are being worn, Dr. Standifer wrote. The unit was located at that time in a valley, and the nearest usable water was in a river a mile and a quarter away. All the Dallas men with the unit were getting long fine, Dr. Standifer said.

Dr. Jess B. Shelmire of Dallas and Dr. Bacon Saunders of Fort Worth have been commissioned in the Volunteer Medical Service Corps authorized by the Council of National Defense.

Commissions in the Medical Officers Reserve Corps were issued to Rex Spencer K. Wood of Waco as First Lieutenant; Howard M. Lanham, Waco, Captain, and F. P. Herff, San Antonio, Captain

Dr. W. L. Crosthwaite, of Waco, has been called to Base Hospital 55, from there he sails for overseas.

